# Dysregulation of sonic hedgehog pathway and pericytes in the brain after lentiviral infection

**DOI:** 10.1186/s12974-019-1463-y

**Published:** 2019-04-13

**Authors:** Diana G. Bohannon, Allen Ko, Adam R. Filipowicz, Marcelo J. Kuroda, Woong-Ki Kim

**Affiliations:** 1 Department of Microbiology and Molecular Cell Biology, Eastern Virginia Medical School, 700 W. Olney Road, Lewis Hall 3174, Norfolk, VA 23501 USA; 2 Division of Immunology, Tulane National Primate Research Center, Covington, Louisiana, USA

**Keywords:** AIDS, Blood-brain barrier, HIV encephalitis, Netrin-1, Pericytes

## Abstract

**Background:**

Impairment of the blood–brain barrier (BBB) has been associated with cognitive decline in many CNS diseases, including HIV-associated neurocognitive disorders (HAND). Recent research suggests an important role for the Sonic hedgehog (Shh) signaling pathway in the maintenance of BBB integrity under both physiological and pathological conditions.

**Methods:**

In the present study, we sought to examine the expression of Shh and its downstream effectors in relation to brain pericytes and BBB integrity in HIV-infected humans and rhesus macaques infected with simian immunodeficiency virus (SIV), an animal model of HIV infection and CNS disease. Cortical brain tissues from uninfected (*n* = 4) and SIV-infected macaques with (SIVE, *n* = 6) or without encephalitis (SIVnoE, *n* = 4) were examined using multi-label, semi-quantitative immunofluorescence microscopy of Shh, netrin-1, tight junction protein zona occludens 1 (ZO1), glial fibrillary acidic protein, CD163, platelet-derived growth factor receptor b (PDGFRB), glucose transporter 1, fibrinogen, and SIV Gag p28.

**Results:**

While Shh presence in the brain persisted during HIV/SIV infection, both netrin-1 immunoreactivity and the size of PDGFRB+ pericytes, a cellular source of netrin-1, were increased around non-lesion-associated vessels in encephalitis compared to uninfected brain or brain without encephalitis, but were completely absent in encephalitic lesions. Hypertrophied pericytes were strongly localized in areas of fibrinogen extravasation and showed the presence of intracellular SIVp28 and HIVp24 by immunofluorescence in all SIV and HIV encephalitis cases examined, respectively.

**Conclusions:**

The lack of pericytes and netrin-1 in encephalitic lesions, in line with downregulation of ZO1 on the fenestrated endothelium, suggests that pericyte loss, despite the strong presence of Shh, contributes to HIV/SIV-induced BBB disruption and neuropathogenesis in HAND.

**Electronic supplementary material:**

The online version of this article (10.1186/s12974-019-1463-y) contains supplementary material, which is available to authorized users.

## Background

The blood–brain barrier (BBB) plays a crucial role in maintaining homeostasis within the brain by regulating molecules entering and exiting the brain parenchyma to prevent neural disruption [1, 2]. The BBB is primarily formed by a single layer of endothelial cells, held together by tight junction proteins (TJPs) and adherent proteins, which defines the luminal space and creates the vessel. Pericytes located abluminally to the endothelial cells surround the vessel and are subsequently surrounded by a ring of astrocytic end-feet, providing structural support and signaling capabilities to the BBB [3–5]. The spaces between the endothelial cells and astrocytic end-feet are filled with the basement membranes, which encase the pericytes and further solidify the BBB. Historically, several neurocognitive disorders, such as Alzheimer’s disease (AD), multiple sclerosis (MS), and HIV-associated neurocognitive disorder (HAND), have been associated with the dysregulation of the BBB resulting in damage to parenchymal neural structures.[6–9]

Recent research has demonstrated the relevance of the Sonic hedgehog (Shh) signaling pathway for maintaining BBB integrity and the loss thereof in brain aging and neurocognitive disorders [10–13]. In the adult brain, astrocytes secrete Shh. Upon secretion, Shh binds and inactivates its receptor Patched-1 (Ptch1), which results in the activation of Smoothened (Smo) and subsequent activation of Gli family transcriptional factors including Gli-1 [10, 14]. While the complete functionality of the Shh signaling pathway is not known, recent studies show that netrin-1 plays an important role in Shh-induced upregulation of TJPs, which are critical for maintaining the selective permeability of the BBB [10, 13, 15, 16].

Despite advances in antiretroviral therapy (ART), HIV-infected patients continue to suffer from HAND, which have been commonly linked to the breakdown of the BBB [17–19]. Previous studies clearly demonstrate the advantages of using a simian immunodeficiency virus (SIV) macaque model to imitate BBB breakdown in the brains of HIV-infected patients [20, 21]. Using this model, we studied changes in Shh signaling and neurovascular supporting cells in the brains of uninfected adult macaques (UI), SIV-infected macaques with no signs of encephalitis (SIVnoE) or with encephalitis (SIVE). A better understanding of the effects of HIV/SIV on the Shh pathway and periendothelial support structures at the glio-vascular interface will help identify potential therapeutic targets to reduce BBB breakdown in HIV-infected patients.

## Methods

### Animal cohorts

A total of 14 adult male rhesus macaques (*Macaca mulatta*) were used in this study. All procedures of this study were approved by the Tulane University Institutional Animal Care and Use Committee (IACUC), and were carried out in accordance with the National Institutes of Health “Guide for the Care and Use of Laboratory Animals”, the recommendations of the Weatherall report, “The use of non-human primates in research,” and the ARRIVE (Animal Research: Reporting In Vivo Experiments) guidelines. All of the animals were housed at the Tulane National Primate Research Center (TNPRC) in accordance with Tulane University’s IACUC regulations and TNPRC endpoint policies. Endpoint policies define 15% weight loss within 2 weeks, unresponsive opportunistic infection, persistent anorexia, severe intractable diarrhea, progressive neurological signs, significant cardiac and/or pulmonary signs, or any other serious illness as adequate terms for euthanasia. This study was conducted on brain tissues from four uninfected, four SIVnoE, and six SIVE animals (Additional file 1: Table S2). Infection groups were intravenously infected with SIVmac251 or SIV0302-2, and all the animals were not perfused at necropsy. Formalin-fixed, paraffin-embedded sections of archival brain tissues from the temporal and occipital cortices were sliced at 5-um thickness. SIVE status was determined by the presence of SIV Gag proteins in the brain, accumulation of macrophages, and the presence of multi-nucleated giant cells (MNGCs).

### Human brain tissues

Formalin-fixed, paraffin-embedded sections of temporal and occipital cortices were obtained from the Manhattan HIV Brain Bank, a member of the National NeuroAIDS Tissue Consortium. A total of 4 HIVE cases with 4 seronegative controls that had been previously described elsewhere were examined [22, 23].

### Immunofluorescence microscopy

Immunofluorescence (IF) microscopy was performed using the primary antibodies listed in Supplementary Table 2. The specificity of primary antibodies was checked in negative controls that omit the primary antibody only. Briefly, sections were incubated at 60 °C overnight before being de-paraffinized and rehydrated in serial xylene and ethanol baths. Pretreatment with Tris- or citrate-based Antigen Unmasking Solution (Vector Laboratories, Burlingame, CA) was done in a microwave (1000 W) for 20 min. After cooling for 20 min, slides were then washed in phosphate-buffered saline (PBS) containing 0.2% fish skin gelatin (FSG) (PBS/FSG). Permeabilization was achieved through incubation with PBS/FSG and 0.1% Triton X-100 at room temperature for 1 h before slides were washed in PBS/FSG. Sections were then blocked with either 5% horse or goat serum for 30 min before the first primary antibody, diluted with PBS/FSG, and was applied at room temperature for 1 h. After PBS/FSG baths, secondary antibodies conjugated to Alexa Fluor 488 or 594 (Molecular Probes, Eugene, OR) were diluted at 1:500 in PBS/FSG and applied for 1 h at room temperature. Subsequent primary and secondary antibodies were applied as described above. Some sections were then counterstained with DAPI for 5 min. After IF staining was complete, sections were washed before being soaked in a quenching solution of 10 mM CuSO_4_ for 45 min. The sections were then washed with distilled water and mounted using a coverslip and Aqua-Mount aqueous mounting medium (Thermo Scientific, Waltham, MA).

A VectaFluor Excel DyLight kit (Vector) was used for Zona Occludin 1 (ZO1) IF staining. All steps described above were followed for this staining, except that horse serum was left on for 1 h instead of 30 min, and the kit’s Amplifier and Reagent were used for 15 and 30 min respectively instead of an Alexa Fluor secondary antibody.

Fluorescent microscopy images were taken with a Zeiss Axio Observer. Z1 fluorescent microscope with a × 20 or × 40 objective. Zeiss AxioVision 4.9.1 edition was used to capture and merge images. Confocal images were taken with a Zeiss 880 Laser scanning confocal microscope with a 100× emersion oil objective. ZenBlack and ZenBlue programs were used to capture and merge images.

### Quantitative analysis

Quantitative analysis was performed using ImageJ and ZenBlack. Intensity density per field was determined through thresholding images to isolate positive immunoreactivity (IR) and using the measure tool to determine the intensity density. Thresholding was achieved by locating areas of each image without specific immunoreactivity, measuring the man pixel intensity (MPI), and averaging the MPI of all images. The average MPI for non-specific staining in the images was then used as a lower limit threshold to subtract background/auto-fluorescence. MPI was determined by thresholding images to isolate positive IR and using the measure tool to find the average intensity of the selected pixels. Percent area was determined by thresholding to obtain only positive IR and using the measure tool to determine what percent of the frame contains positive staining. Total intensity was obtained by adding the pixel intensity of each pixel within the selected area into a raw score demonstrating the IR in that area. The percent of vessels demonstrating fibrinogen extravasation was determined by running linear plot profiles on the green and red channels of individual vessels captured at × 400 magnification and graphing them in GraphPad as dual overlay histograms. The histograms were then analyzed to determine whether the fibrinogen was above background levels outside of the two primary glucose transporter 1 (GLUT1) peaks; vessels that displayed this phenotype were considered to be extravasated. The number of extravasated vessels was divided by the total number of vessels counted to calculate the percentage of vessels extravasated. Confocal Z-stack images were analyzed through a ZenBlack co-localization experiment. Thresholds for PDGFRB and SIVp28 were set to isolate positive IF, and the program determined the degree of co-localization between the channels. Finally, each SIVE animal was given a pathological disease score based upon the average number of lesions per centimeter square of white matter determined by observation of 15 sections per animal.

All quantifications were originally carried out in gray and white matter separately; however, while some differences in the amount of IR were seen between the two tissue types, there were no significant differences between infection groups. As such, gray and white matter were combined to form a single data point for each animal. Additionally, no lesion-associated vessels were taken into consideration during quantification unless specifically stated as such.

Pericyte thickness was calculated by measuring the total area of the pericyte coverage, excluding endothelial and luminal areas, and dividing the pericyte area by vessel area. This calculation allows for the measurement of pericyte thickness which is normalized to exclude variation caused by the size of vessels. Pericytes were determined to be hypertrophied when they were greater than 150% luminal area by a receiver operating characteristic curve analysis.

A non-lesion-associated, normal-appearing small vessel is defined as a vessel less than 15 μm in luminal diameter showing endothelial continuity with no signs of extra-luminal thrombosis or perivascular accumulation of white blood cells. In the interest of ensuring that all vessels were nearly horizontal cross-sections, no single luminal radius was permitted to be more than twice the length of the shortest luminal radius. Lesions were identified through both SIVp28+ staining and accumulation of no less than eight CD68+ macrophages. All lesions in images are circled with dotted lines.

### Statistical analysis

GraphPad Prism 7.2 was used to graph data and analyze its significance. A one-way ANOVA in conjunction with a Dunn’s multiple comparison test was performed to determine the significance of numerical comparisons between study groups. A two-tailed paired *t* test was used to determine significant differences between types of vessels within the same animal. * denotes *p* < 0.05, ** denotes *p* < 0.01, *** denotes *p* < 0.001, **** denotes *p* < 0.0001. All error bars denote standard deviation (SD).

## Results

### Shh expression during SIV infection

**Fig. 1:**
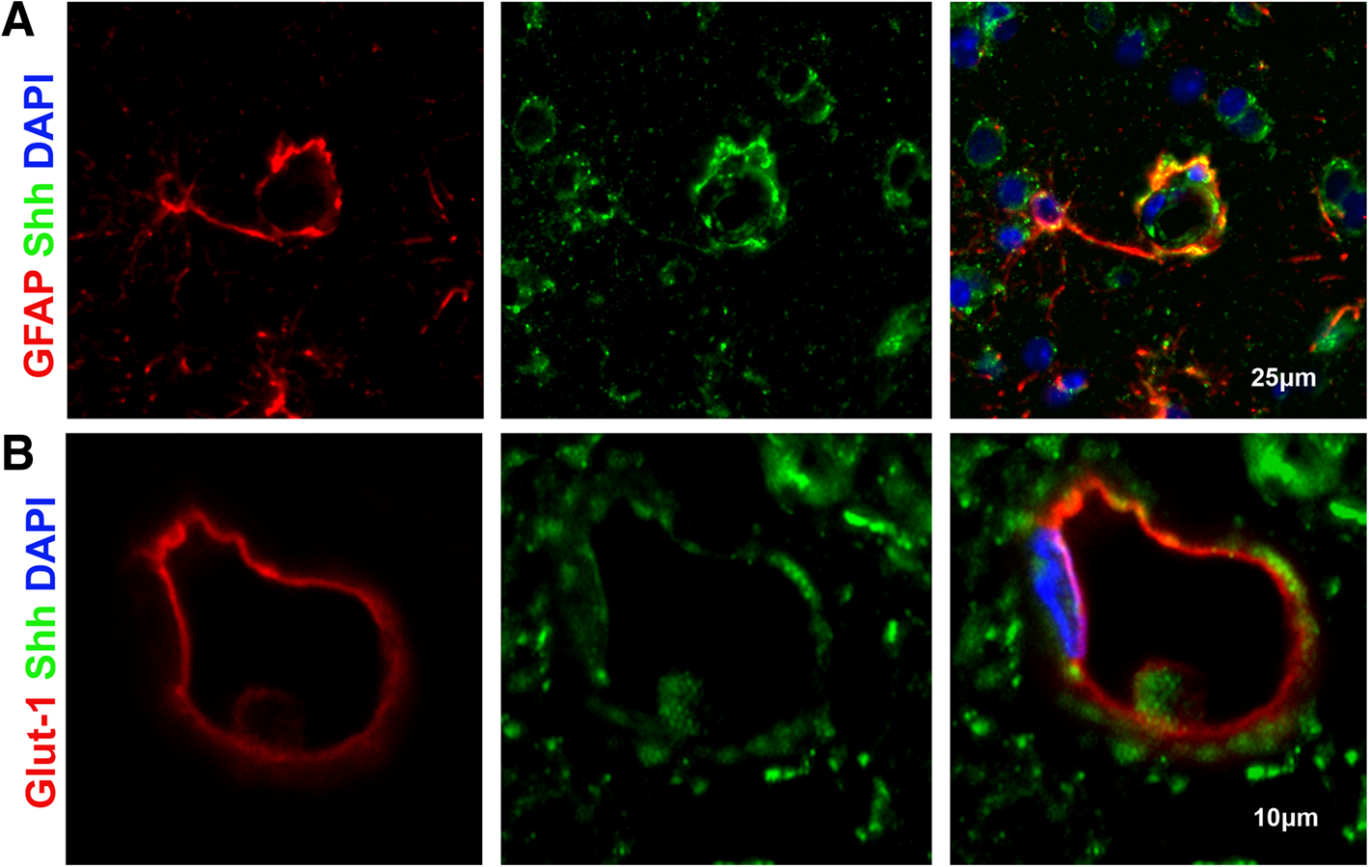
Shh found on astrocytic end-feet and near endothelial cells within the glio-vascular unit triple-label IF imaging of uninfected animals for GFAP (red), Shh (green), and DAPI (blue) confirmed the presence of Shh on astrocytic end-feet (**a**). Likewise, IF imaging of uninfected animals for GLUT1 (red), Shh (green), and DAPI (blue) showed Shh located adjacent to endothelial cells **b**

In this study, we sought to investigate changes in the Shh pathway at the BBB following SIV infection. First, we examined Shh protein expression in the brains of uninfected, SIVnoE and SIVE macaques by semi-quantitative IF microscopy (Supplementary Table 1). Cells within the glio-vascular interface that demonstrated an association with Shh IR in uninfected macaques were identified as astrocytes and endothelial cells via triple immunofluorescence staining for astrocyte marker glial fibrillary acidic protein (GFAP), endothelial cell marker GLUT1, and Shh (Fig. 1). In addition to neurons, these cells are known to be important contributors to the Shh pathway as astrocytes are considered a primary cellular source of Shh, and endothelial cells act as its primary site of action [10]. Shh IR was found in astrocytic processes and end-feet, and in close proximity to the endothelium (Fig. 1).

**Fig. 2:**
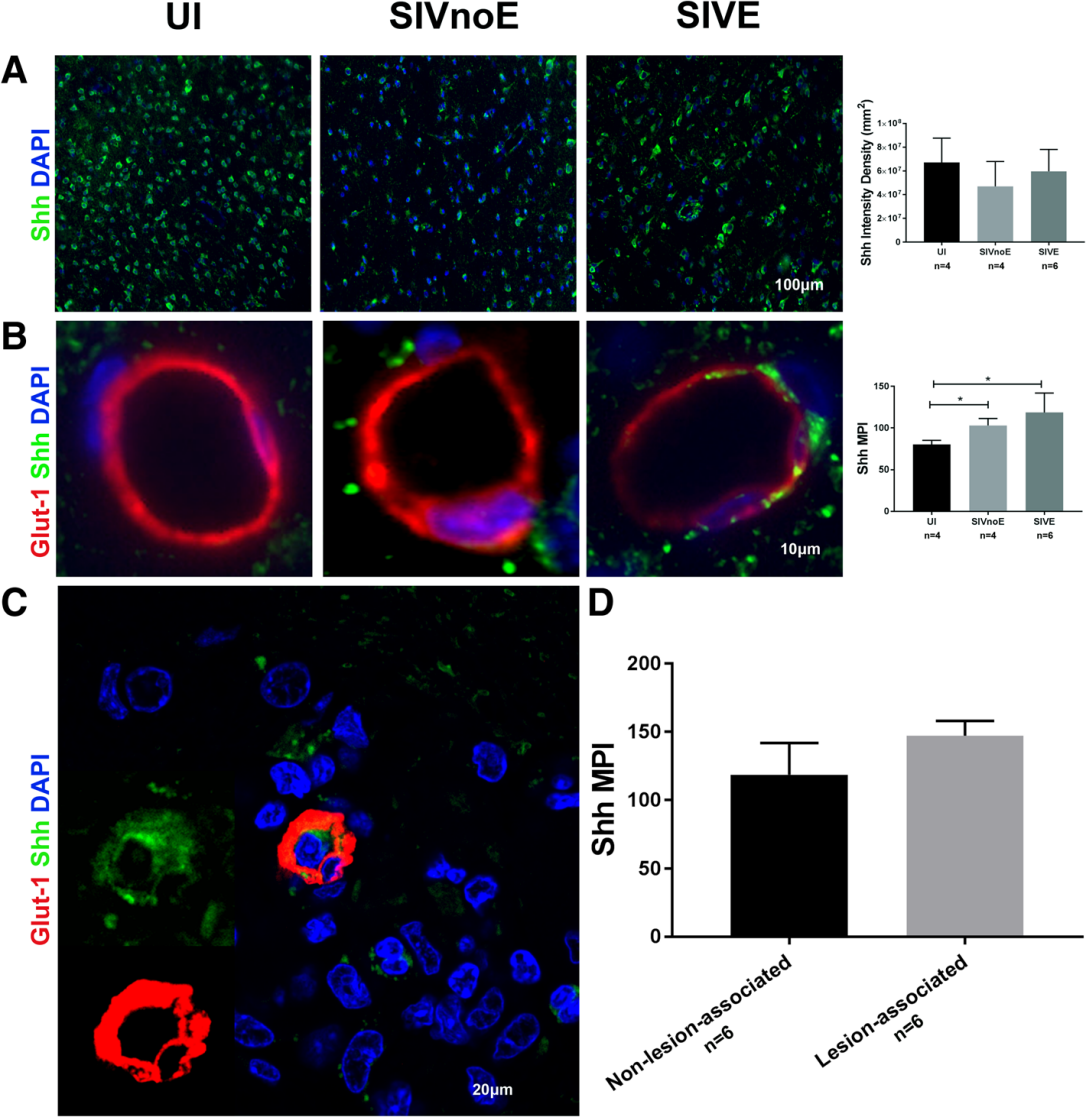
Dysregulation of Shh with SIV infection double-label IF for Shh (green) and DAPI (blue) shows no significant difference between groups **a**. Triple-label IF of GLUT1 (red), Shh (green), and DAPI (blue) shows an increase in the MPI of Shh near the endothelium of both infection groups when compared to the control group (**b**). Likewise, IF imaging of Shh (green), GLUT1 (red), and DAPI (blue) in a SIVE lesion shows consistently high levels of Shh near the lesion-associated endothelium **c**. No significant difference was found in Shh levels between non-lesion-associated vessels and lesion-associated vessels in SIVE **d**. Twelve lesions were used from each SIVE animal (*n* = 6), and Shh MFI was compared between non-lesion and lesion-associated vessels in each animal. Lesions are circled with white dotted lines (**c**). Error bars denote SD

Overall, Shh IR did not differ significantly between the three groups (Fig. 2a) either for white or gray matter (data not shown). As there was no statistically significant difference in white and gray matter between the groups, no separate analyses of the gray matter and the white matter were made. Since astrogliosis was observed in all SIV-infected macaques (Additional file 1: Table S1), we initially expected an increase in Shh IR in astrocytes. However, enhanced Shh was only noted in proximity to the endothelium of SIV-infected monkeys (Fig. 2b). Interestingly, Shh levels remained high adjacent to lesion-associated vessels in SIVE animals suggesting its continued availability during lesion formation (Fig. 2c, d). Shh IR persists during SIV infection and SIVE lesion formation, suggesting any breakdown in the signaling cascade is not likely due to lack of available Shh proteins (Fig. 2).

### Dysregulation of netrin-1 and PDGFRB during SIV infection

**Fig. 3:**
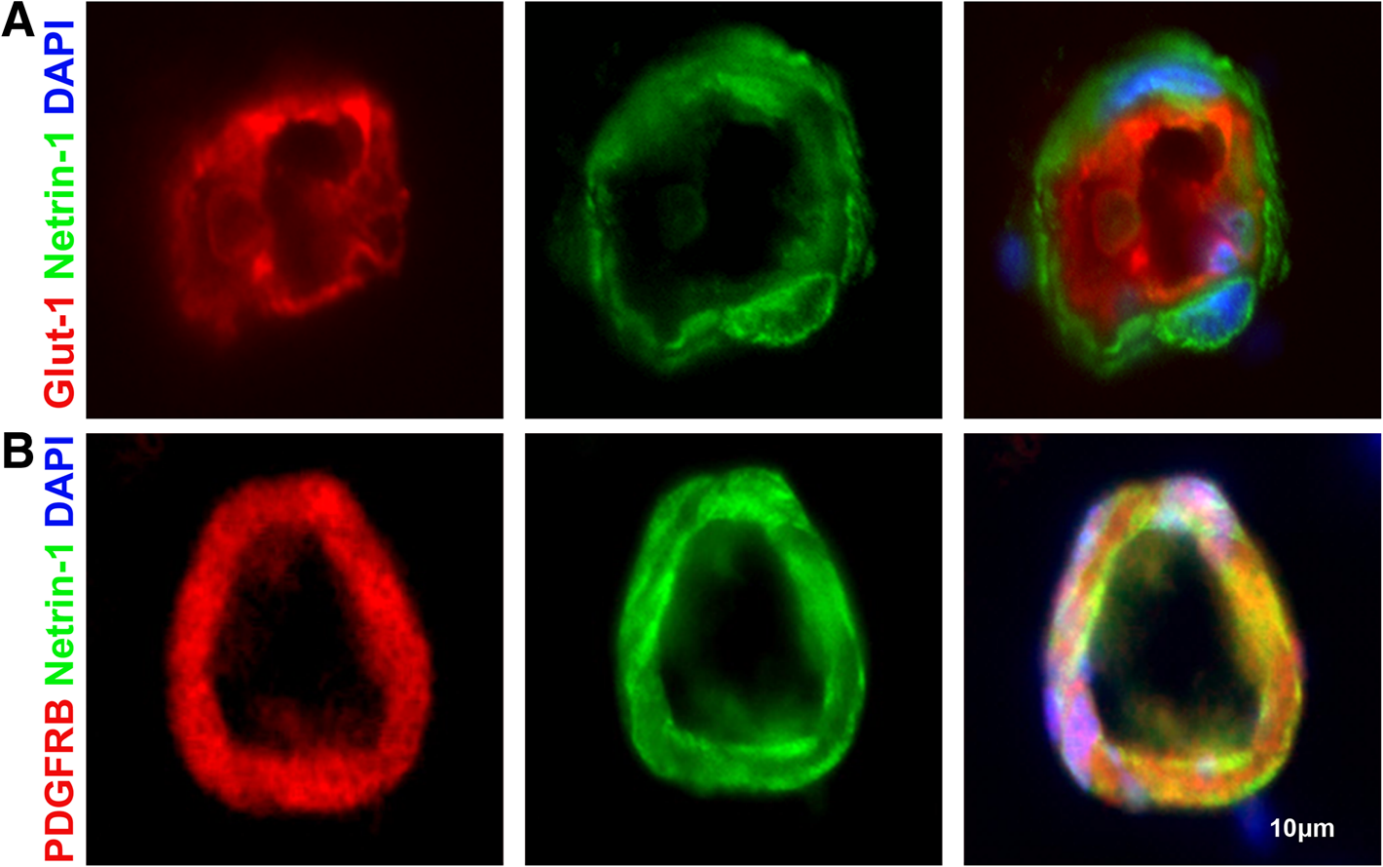
Netrin-1 found in pericytes within the neurovascular niche triple IF staining of uninfected animals for GLUT1 (red), netrin-1 (green), and DAPI (blue) shows netrin-1 surrounding the endothelium (**a**). Triple IF staining of uninfected animals for PDGFRB (red), netrin-1 (green), and DAPI (blue) shows netrin-1 co-localizing with pericytes at the glio-vascular interface **b**

**Fig. 4:**
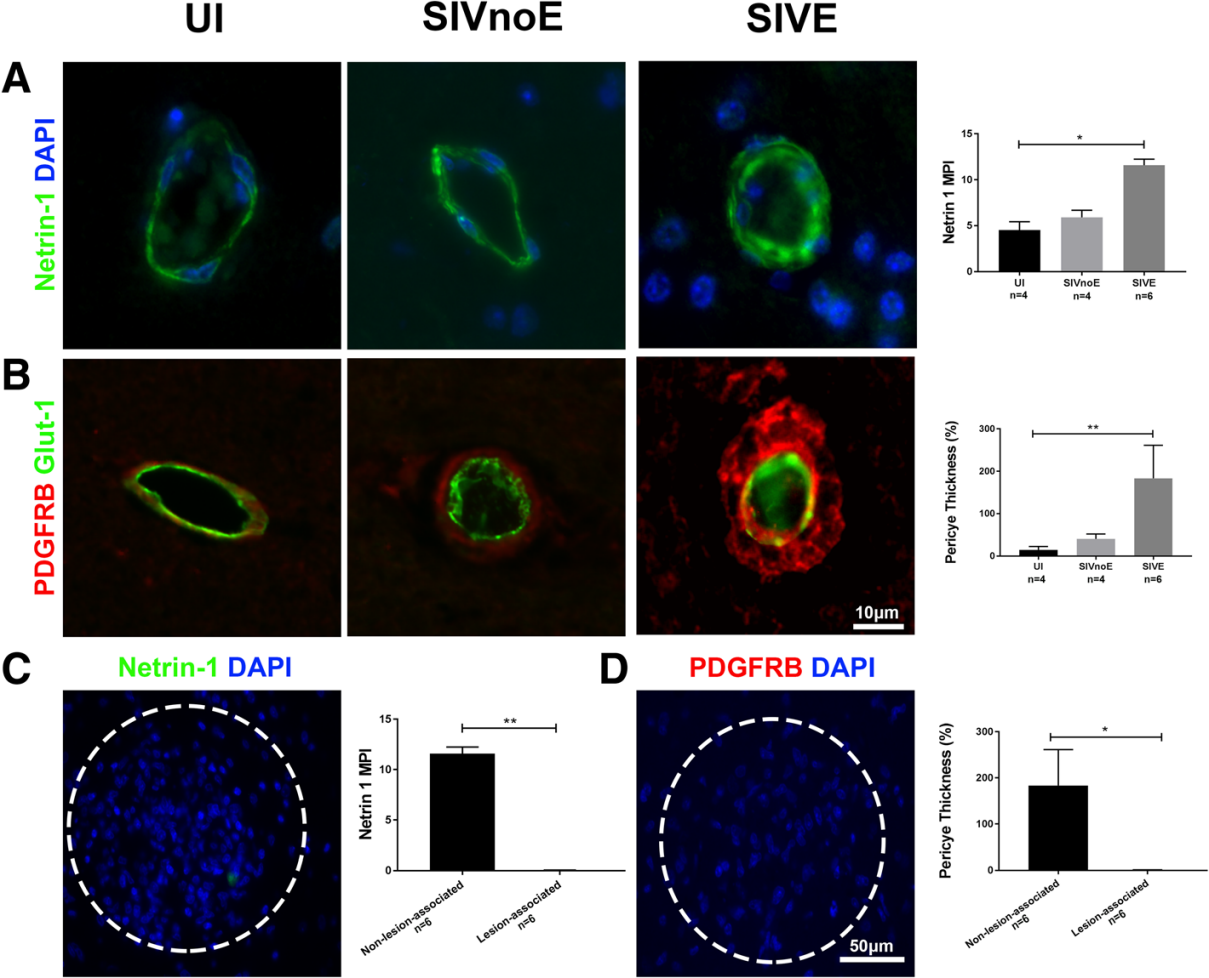
Pericytes and netrin-1 dysregulated with SIV infection IF imaging of netrin-1 (green) and DAPI (blue) show an increase in netrin-1 MPI in SIVE animals (**a**). Double IF staining of PDGFRB (red) and GLUT1 (green) displays increased pericyte thickness in SIVE animals compared to uninfected (**b**). Double IF staining for PDGFRB (red), and DAPI (blue), shows that PDGFRB is eliminated in SIVE lesions (**d**). Likewise, staining for netrin-1 (green), and DAPI (blue), shows a similar elimination of netrin-1 in SIVE lesions **c**. Twelve lesions were used from each SIVE animal (*n* = 6) and PDGFRB area (**c**) or netrin-1 MPI (**d**) was compared between non-lesion and lesion-associated vessels in each animal. Lesions are circled within white dotted lines (**c**, **d**). Error bars denote SD

Considering the persistent presence of Shh at the BBB after SIV infection, we investigated whether downstream effectors of the pathway were subject to dysregulation. Netrin-1 is known as an important downstream effector of the Shh signaling cascade and best known for its role in promoting TJPs and maintaining the stability of the BBB [13, 24]. Building upon published literature that strongly suggests the presence of netrin-1 in endothelial cells [13], we performed triple-label immunofluorescence imaging for GLUT1 and netrin-1 in uninfected macaque cortical brain tissue (Fig. 3a). To our surprise, there was no co-presence of netrin-1 and GLUT1 IR. However, subsequent staining for PDGFRB and netrin-1 showed co-localization, indicating pericytes as the major resident cellular source of netrin-1 at the BBB (Fig. 3b). Further investigation of netrin-1 showed a significant increase in netrin-1 MPI in SIVE animals when compared to the uninfected (Fig. 4a). In support of the idea that netrin-1 downstream signaling is maintained after infection, we found no significant difference in ZO1 IR associated with normal-appearing small vessels (see “Methods” section for definition) across groups (Additional file 1: Figure S1b). Interestingly, these findings were accompanied by the observation that pericyte thickness was significantly increased in SIVE animals when compared to the uninfected (Fig. 4b). Since both pericyte thickness and netrin-1 IR were increased in SIVE animals and ZO1 IR was maintained, we initially expected to find the continued presence of pericytes in lesion-associated vessels. However, neither netrin-1 nor PDGFRB IR was found around lesion-associated vessels (Fig. 4c, d), nor was there detectable IR for ZO1 (data not shown). The absence of pericytes in the lesion was further confirmed using a second pericyte marker CD146, which was present around non-lesion-associated vessels, but lacking in lesions (Figure S1e). While netrin-1 IR and pericyte thickness are increased during SIV infection with the development of SIVE, both are absent in lesions suggesting potential disruption of the Shh signaling pathway in relation to lesion formation (Fig. 4).

### Increase in pericyte thickness due to hypertrophy, not proliferation

**Fig. 5:**
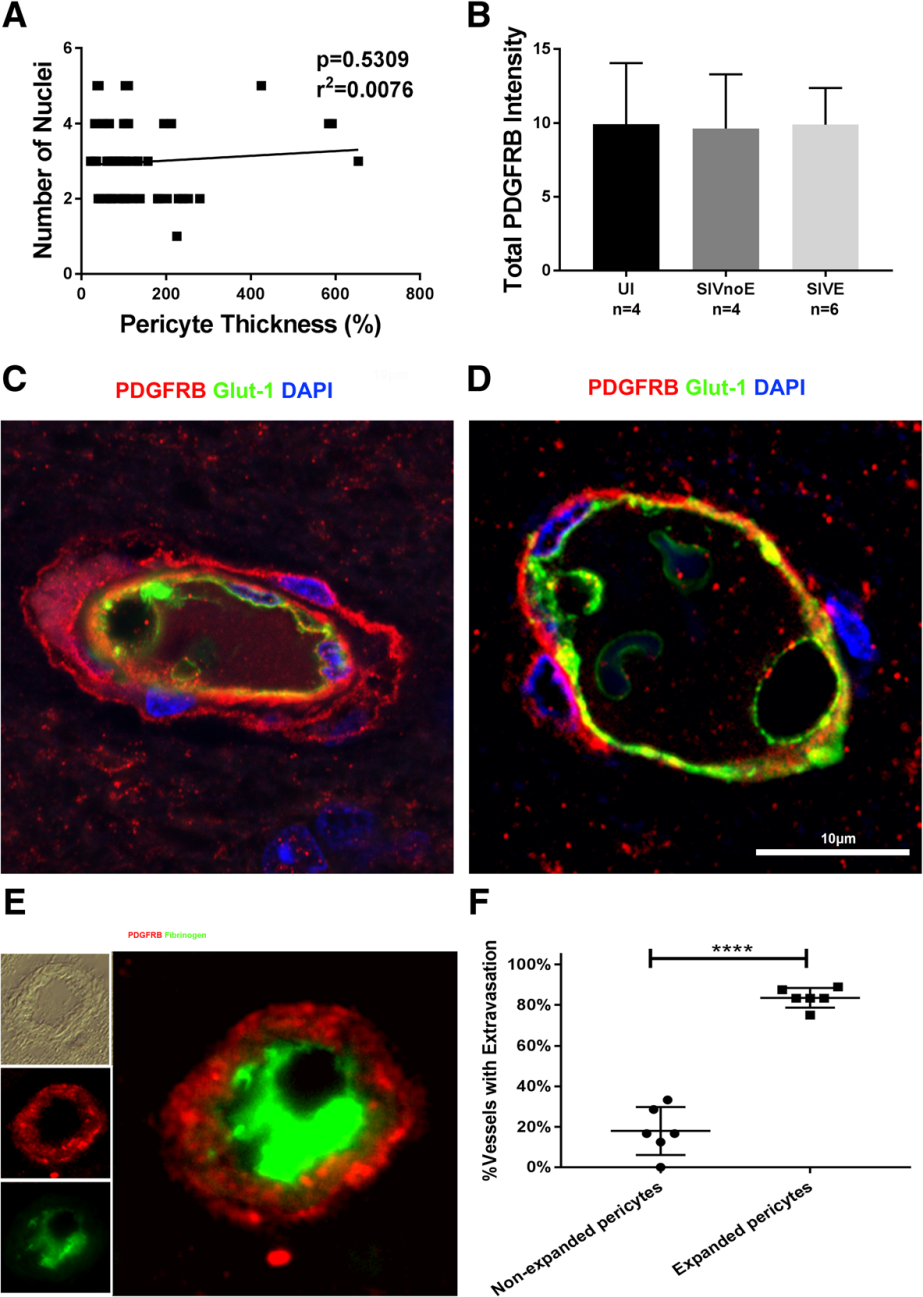
Changes in pericyte thickness of SIVE brain are due to cellular changes, not pericyte number. Pericyte thickness graphed against the number of pericytic nuclei shows no significant trend between the number of pericytes and their thickness around the endothelium of SIVE infected animals (**a**). Total PDGFRB intensity shows no change in PDGFRB expression between study groups despite the significant difference in pericyte area (**b**). Triple-label IF for PDGFRB (red), GLUT1 (green), and DAPI (blue) of an expanded pericyte (**c**) and a non-expanded pericyte (**d**) show the cellular differences between two pericytes in the same SIVE animal (10A067). Using double-label IF for PDGFRB (red) and fibrinogen (green), we were able to determine that 80% of vessels surrounded by enlarged pericytes showed signs of fibrinogen extravasation (**e**, **f**). Error bar denotes SD. Ten vessels were randomly chosen from each SIVE animal (*n* = 6); each point on the graph **a** denotes a single vessel. No significant difference was found in **a** or **b**. A paired *t* test was used to compare between samples in **f**

To help clarify this morphological change of pericytes after SIV infection, we first investigated whether the increase in pericyte coverage around the endothelium was due to an increase in the number of pericytes surrounding these vessels or the size of each pericyte. By plotting the number of pericytes around each vessel against the thickness of pericyte coverage, we were able to determine that there was no significant correlation between the two (Fig. 5a). This, in conjunction with our finding that total PDGFRB IR did not change between study groups (Fig. 5b) despite the observed change in pericyte thickness (Fig. 4b), lead us to the conclusion that pericyte size, not number, was responsible for pericyte thickening. This was further confirmed through confocal microscopy. High magnification images (× 1000) were taken of endothelial cell coverage by both hypertrophied and non-hypertrophied pericytes on the same section of SIVE cortical brain tissue to show the visual difference between the two (Fig. 5c, d). This data suggests that thickened pericyte coverage is likely due to damage-induced hypertrophy of the cells, not a proliferation-based response mechanism to infection (Fig. 5).

### Hypertrophied pericytes are associated with BBB breakdown and SIV infection

To further evaluate the connection between pericyte hypertrophy and SIV infection, we studied the breakdown of the BBB. Fibrinogen extravasation is an accepted method of measuring BBB disruption in post-mortem samples by visualizing endogenous blood plasma proteins [25]. First, we measured the percent of vessels demonstrating fibrinogen extravasation (*n* = 500 per animal) and found a significant increase in SIVE animals when compared to both uninfected and SIVnoE groups (Figure S1c). These data were further visualized by dual histogram overlays in which any fibrinogen IF (red) that occurs outside of the two primary GLUT1 IF (green) peaks on the *x*-axis is considered to be extravasated fibrinogen (Figure S1d). In addition, we found that 80% of vessels with hypertrophied pericyte coverage (*n* = 60) showed fibrinogen extravasation, while it occurred in less than 20% of vessels covered by non-hypertrophied pericytes (*n* = 60) (Fig. 5e, f). This demonstrates an association between areas of BBB breakdown and hypertrophied pericyte coverage.

**Fig. 6:**
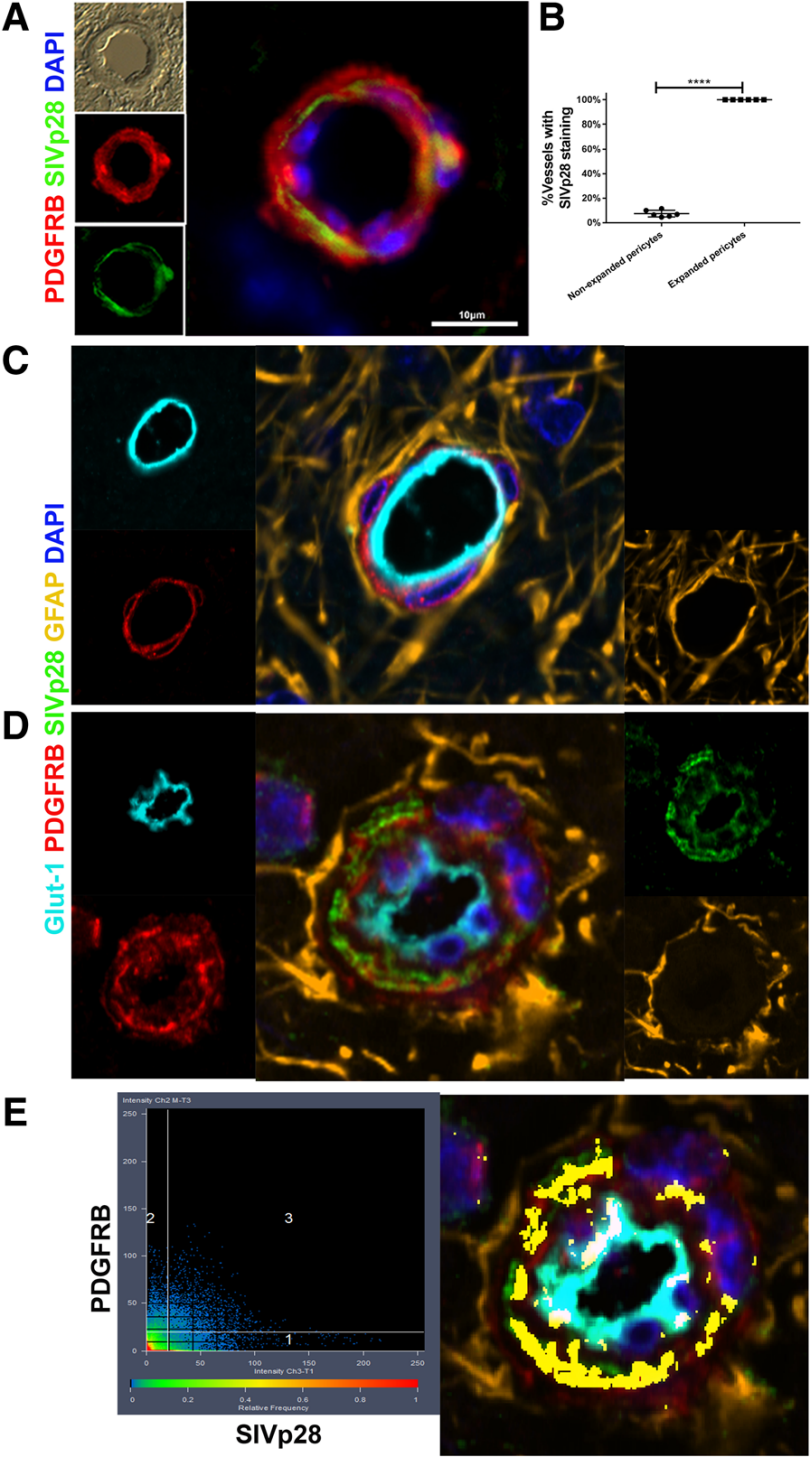
Enlarged pericytes highly localized to areas of fibrinogen extravasation and pericyte infection IF imaging for PDGFRB (red), SIVp28 (green), and DAPI (blue) **a** of 10 vessels with and 10 without enlarged pericyte coverage for each SIVE animal (*n* = 6) showed that 100% of the enlarged pericytes (*n* = 60) had localized SIVp28+ staining, while only 8% of the non-enlarged pericytes (*n* = 60) showed positive staining (**b**). Five-color IF staining for GFAP (gold), PDGFRB (red), GLUT1 (aqua), SIVp28 (green), and DAPI (dark blue) show the major cellular components of the glio-vascular interface and how they localize with SIVp28 viral protein marker in the non-hypertrophied pericyte of an uninfected animal (11A014) and the hypertrophied pericyte of an SIVE animal (11A554) (**c**, **d**). This demonstrates the presence of SIVp28 primarily in the pericytes and endothelial cells (**d**). Presence of SIVp28+ staining within pericytes was confirmed via a confocal microscopy Z-stack of a 20-μm-thick section using 0.25 μm increments analyzed with a ZenBlack co-localization test in which points in quadrant 3 and highlighted in yellow are considered to be co-localized between PDGFRB and SIVp28 (**e**). A paired *t* test was used to determine significance between groups in **b**

To determine how this may relate to SIV infection, we studied the association between hypertrophied pericytes and SIVp28 protein IR in SIVE. In examining 60 hypertrophied and 60 non-hypertrophied pericytes from SIVE animals, we found that all 60 vessels with hypertrophied pericytes showed SIVp28 localization to the pericytes, while only 8% of non-hypertrophied pericytes demonstrated SIVp28 localization (Fig. 6a, b). To determine whether hypertrophied pericytes contained SIV Gag p28, we utilized a five-color immunofluorescence staining to visualize the major cell components within the glio-vascular unit. A side-by-side comparison of a non-hypertrophied vessel from an uninfected animal and a hypertrophied vessel from a SIVE animal shows marked differences between the vessel phenotypes particularly in pericyte organization (Fig. 6c, d). A ZenBlack co-localization test of confocal Z-stack images of 20-μm-thick sections displaying astrocytes, pericytes, endothelial cells, their nuclei, and SIVp28 protein at the BBB showed that these hypertrophied pericytes were positive for SIV Gag p28 (Fig. 6e). In support of this, in vitro studies showing the capability of HIV-1 to enter and replicate within human brain pericytes suggest that HIV-1 is capable of infecting these cells [26, 27].

### Shh persistence and infection of hypertrophied pericytes confirmed in human cortical brain tissue

**Fig. 7:**
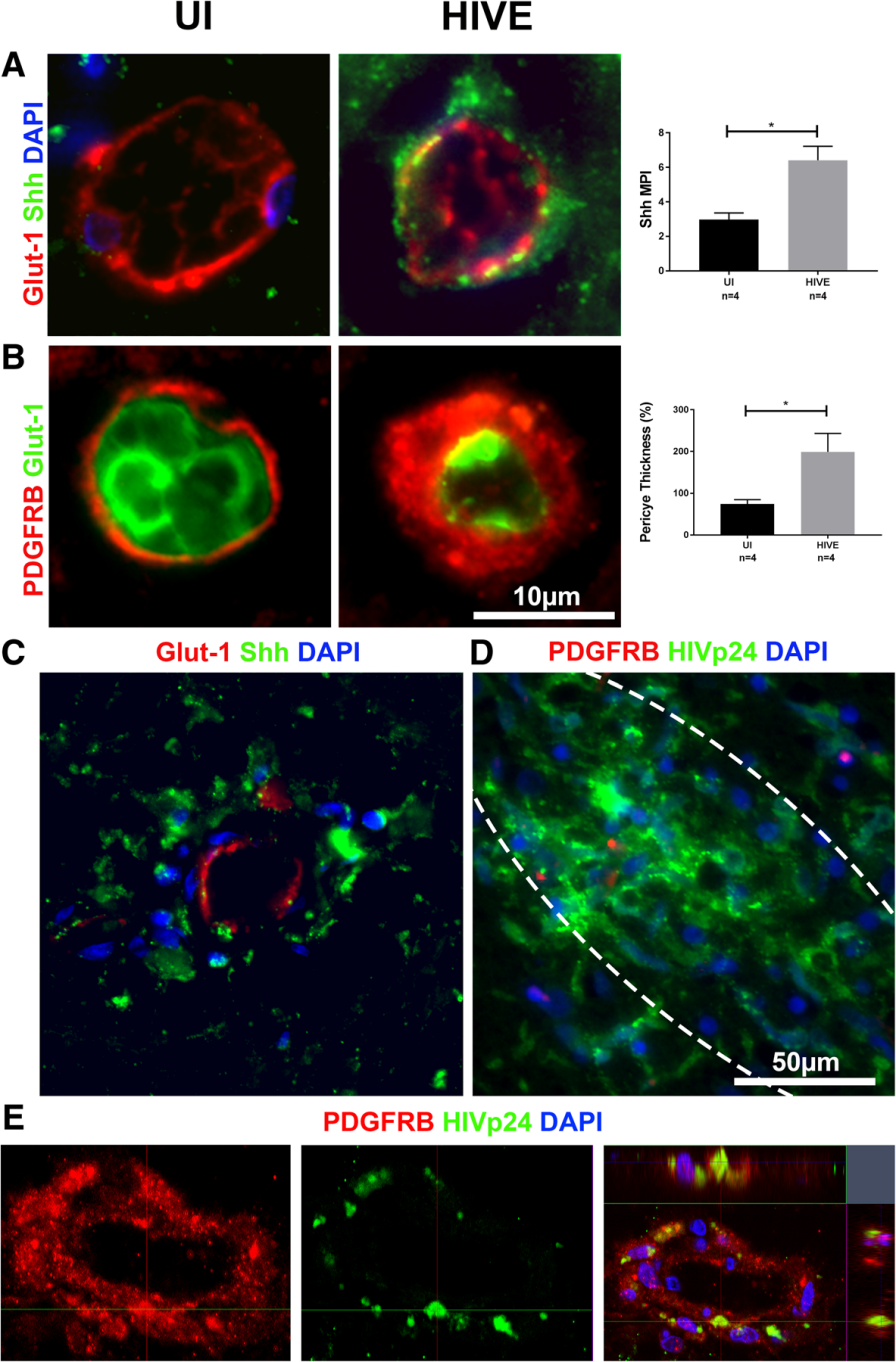
Findings in rhesus macaques confirmed in human tissue triple IF for Shh (green), GLUT1 (red), and DAPI (blue) show increased IR of Shh in association with the vessels of HIVE patients when compared to UI **a** and remained high in lesion-associated vessels (**c**). Likewise, double IF for GLUT1 (green) and PDGFRB (red) shows an increase in pericyte thickness in HIVE patients (**b**). Despite this increase, however, triple IF for HIVp24 (green), PDGFRB (red), and DAPI (blue) demonstrates a marked lack of PDGFRB+ pericytes in lesions (**d**). Further investigation of the same IF stain shows HIV Gag p24 localized to hypertrophied pericytes. The presence of HIVp24 within human brain pericytes was confirmed via a confocal microscopy Z-stack using 0.25 μm increments and an orthogonal view representation **e**. Lesions are circled with white dotted lines (**d**). A paired *t* test was used to determine significance between groups in **a** and **b**

To confirm that our findings in rhesus macaques hold clinical relevance for humans infected with HIV, we examined four uninfected and four HIVE cases. Like with the animal model, there was a significant difference in the amount of Shh localized to the endothelium between uninfected and HIVE groups, which persisted within lesion-associated vessels (Fig. 7a, c). Additionally, we found a significant increase in pericyte thickness in subjects with HIVE compared to uninfected, but again, no signs of PDGFRB IR around lesion-associated vessels (Fig. 7b, d). Z-stacking was performed to demonstrate the presence of HIV p24 antigen within hypertrophied pericytes in the brains of HIVE patients (Fig. 7e). In support of our earlier findings in SIV-infected rhesus macaques, this result demonstrates that pericytes containing HIVp24 become hypertrophied in the glio-vascular unit of HIVE patients despite consistent availability of the Shh protein.

### Disease severity is strongly correlated with dysregulation of ZO1, pericyte morphology, and BBB permeability

**Fig. 8:**
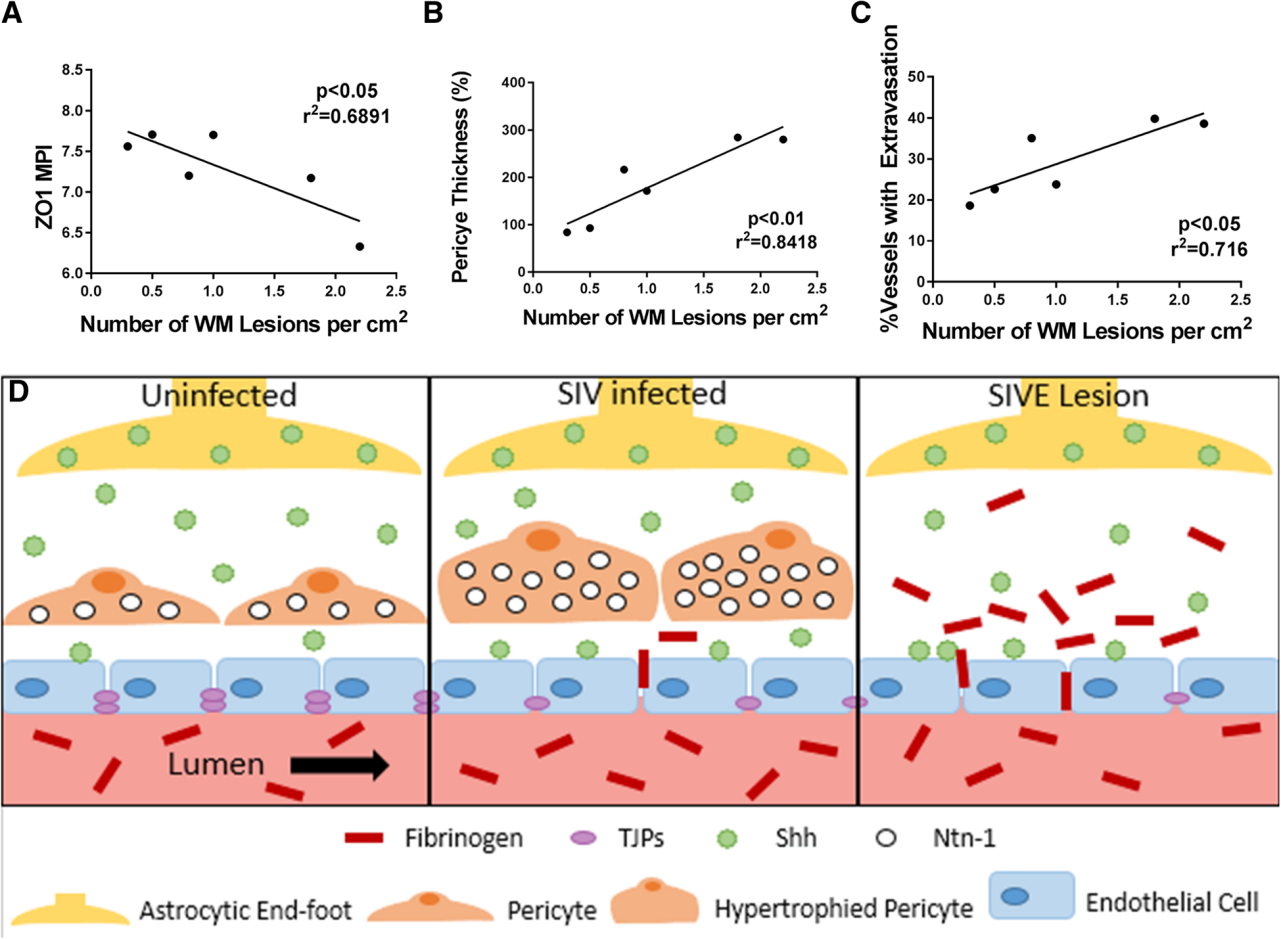
BBB instability, pericyte thickness, and ZO1 loss correlate with the severity of SIVE. The severity of encephalitis was approximated by counting the average number of lesions per square cm of white matter tissue (*n* = 15 slides per SIVE animal). Each point on the graph designates the mean value of one SIVE animal (**a–c**). ZO1 MPI decreased in accordance with the lesion number (*p* < 0.05, *r*^2^ = 0.689) (**a**). The average pericyte area increases with lesion number (*p* < 0.01, *r*^2^ = 0.842) (**b**). The percent of vessels demonstrating fibrinogen extravasation increased with lesion number (*p* < 0.05, *r*^2^ = 0.716) (**c**). Lines demonstrate linear regressions (**a–c**). An illustrative representation of changes with the glio-vascular unit through disease progression depicts a section of the BBB in uninfected, SIV-infected, and SIVE animals (**d**). Uninfected animals have an intact endothelium with TJPs between endothelial cells, non-hypertrophied pericytes, fibrinogen confined within the lumen of brain vessels, Shh localized primarily in the open space pace between the endothelial cells and the astrocyte endfeet (**d**), left panel. SIV-infected animals show a partially fenestrated endothelium missing some TJPs, hypertrophied pericytes with increased netrin-1, some fibrinogen escaping the lumen, and Shh more heavily localized to the endothelium (**d**), middle panel. Within the lesion, encephalitic animals have a highly fenestrated endothelial layer with an almost complete loss of TJPs, high levels of fibrinogen extravasation, a complete loss of pericytes and netrin-1, and a high proportion of Shh localized to the endothelium (**d**), right panel

To determine whether disease progression played a role in the severity of changes seen, we calculated a disease score for each SIVE animal by calculating the number of lesions per centimeter square of white matter. We found that ZO1 expression is negatively correlated with the severity of SIVE (Fig. 8a). Pericyte thickness and fibrinogen extravasation are both strongly positively correlated with the severity of SIVE, suggesting that hypertrophied pericytes and increased frequency of BBB disruption may be associated with SIVE progression in SIV-infected macaques (Fig. 8b, c). Our model suggests that hypertrophied pericytes may be involved in the initiation of BBB destabilization in SIV-infected animals, and the loss of pericytes further disrupts the BBB stability (Fig. 8d).

## Discussion

A recent study in a humanized mouse model suggests a decrease in Shh production with HIV infection [28]. However, our study found no significant changes in overall Shh production and instead an increase in Shh localized to the endothelium within the lesion and to non-lesion-associated vessels in SIV-infected rhesus macaques. The use of different models and stages of infection may account for this difference. We also noted the absence of PDGFRB+ pericytes and netrin-1 within HIVE/SIVE lesions suggesting that the loss of pericytes may play a role in HIV/SIV-induced BBB breakdown and lesion formation. Our findings may help to direct future therapeutic studies surrounding pericyte damage and loss in HIV/SIV infection and underline the importance of pericyte preservation and Shh downstream effector stability in maintaining the selective permeability of the BBB during HIV/SIV infection.

Shh has been noted as a significant factor in damage repair across multiple neurocognitive diseases and disorders including MS and ischemic brain injury, as well as HAND. Shh has been seen in astrocytes in all stages of MS lesions as well as in normal appearing white matter within the MS brain, but high levels of Shh + macrophages tend to be present in demyelinating lesions and less so in remyelinating lesions, which primarily house Shh + axons [10, 29, 30]. A similar increase in Shh is found within areas of ischemic stroke, and an inhibition of Smo has been shown to prevent natural recovery, while eliminating Shh has been shown to reduce the number of regionally produced Olig2+ cells during the post-ischemia recuperation process [11, 31–33]. These studies and many others show the reparative abilities of Shh within the glio-vascular unit, but recent studies have suggested that HIV is capable of disrupting the production of Shh preventing its continued maintenance and repair of the BBB [28]. Despite the presence of Shh in the brain of SIV-infected macaques, the loss of netrin-1 within SIVE lesions suggests that a downstream disruption of the Shh pathway may play a role in BBB breakdown. Evidence suggests that the loss of netrin-1 is likely due to the loss of its host cell, the pericyte, which become hypertrophied in association with HIV/SIV infection of the brain, and is absent in encephalitic lesions.

Pericyte dysfunction is not unique to HIV/SIV infection. Previous studies suggest that pericyte dysfunction may play a key role in diseases like AD, cancer, seizure disorders, and even natural aging. Pericytes have been a key target in AD research due to their ability to traffic amyloid beta deposits in the brain back into the bloodstream for removal by other organs, making their dysfunction likely to result in AD-like brain pathology [34–39]. Recently, high numbers of cells expressing NG2, a commonly used pericyte marker, have been seen within high-grade choroid plexus tumors suggesting a potential role for pericytes in cancer [40]. Pericytes have also been suggested to be involved with the cerebrovascular rearrangement seen in seizure patients [41]. Changes in pericytes during normal aging have been linked to localized hypoxia as well as BBB leakage and an increase in neurotoxicity within the brain [42]. Studies with animal models of traumatic brain injury suggest that pericytes play a key role in the construction and repair of the brain’s microvascular structures [43, 44]. Although studies investigating the effects of HIV/SIV on brain pericytes reported an overall decrease in pericyte coverage [45–47], we found that pericytes around many of the vessels in SIVE animals are becoming hypertrophied. This suggests that they may be undergoing functional changes or damage prior to being eliminated upon lesion formation. While no ART-treated animals were investigated in this study, Fig. 8 shows that both pericyte thickness and fibrinogen extravasation are positively correlated with the severity of SIVE suggesting that pericyte hypertrophy may continue to contribute to BBB disruption even in virally suppressed individuals.

Further research is needed to fully understand changes in the Shh pathway in the adult brain during HIV/SIV infection and the effect on the BBB. Additionally, further studies are needed in order to determine the cause and effect of pericyte hypertrophy in adult macaques with SIVE. Despite suggestions concerning Shh as a potential therapeutic target for HIV-associated BBB breakdown [28], the constitutive expression and increased localization of Shh to the endothelium in vivo calls into question its value as a therapeutic agent. Disruption of downstream effectors of the Shh pathway, such as netrin-1, may still play a key role in the dysregulation of the BBB during HIV/SIV infection due to SIV-associated pericyte dysfunction, which may result in a disruption of this and other key pathways at the glio-vascular interface.

## Conclusions

In conclusion, the current study provided evidence for brain pericyte hypertrophy and loss, concomitant with disruption of BBB, in the brains of SIV-infected macaques and HIV-infected patients. Further research on pericyte dysfunction with HIV/SIV infection is needed to determine its role in the dysregulation of the Shh pathway and HAND as well as to identify any links to other neurocognitive disorders and diseases.

## Additional files

Additional file 1: **Table S1.** Animals recruited in this study. **Table S2.** Primary antibodies used in study. **Fig. S1.** Astrocytosis and evidence of BBB breakdown observed with SIV infection Double IF staining for GFAP (red) and DAPI (blue) shows a significant increase in astrocyte coverage with SIV infection (**a**). Double IF staining for ZO1 (green) and DAPI (blue) shows a trending decrease in ZO1 expression with infection, but no significant difference (**b**). Triple-label IF of fibrinogen (red), Glut-1 (green), and DAPI (blue), shows a significant increase in the percent of vessels showing fibrinogen extravasation in SIVE animals when compared to uninfected (**c**). Linear analysis of MFI for both GLUT1 (green) and fibrinogen (red) provides an overlaid histogram view of the vessels in Figure S1c (**d**). Fibrinogen (red) that occurs outside the x-axis bounds of the two main GLUT1 (green) peaks is considered to be extravasated fibrinogen (**d**). Error bars indicate SD. Triple IF staining for vascular GLUT1 (green), pericyte CD146 (red) and nuclear DAPI (blue) shows a non-lesion-associated vessel (left) with pericyte coverage and a lesion-associated vessel (right) without pericyte coverage (**e**). (Docx 294 kb)

## Abbreviations

AD: Alzheimer’s disease
ART: Antiretroviral therapy
BBB: Blood–brain barrier
CNS: Central nervous system
FSG: Fish skin gelatin
GFAP: Glial fibrillary acidic protein
GLUT1: Glucose transporter 1
HAND: HIV-associated neurocognitive disorders
IACUC: Institutional Animal Care and Use Committee
IF: Immunofluorescence
IR: Immunoreactivity
MNGC: Multinucleated giant cell
MPI: Mean pixel intensity
MS: Multiple sclerosis
PBS: Phosphate-buffered saline
PDGFRB: Platelet-derived growth factor receptor beta
Ptch1: Patched 1
Shh: Sonic hedgehog
SIV: Simian immunodeficiency virus
SIVE SIV: Encephalitis
SIVnoE: SIV-infected without encephalitis
Smo: Smoothened
TJP: Tight junction protein
TNPRC: Tulane National Primate Research Center
UI: Uninfected
ZO1: Zona occludens 1
