# Supplementary material for: Dysregulation of sonic hedgehog pathway and pericytes in the brain after lentiviral infection
Source: J Neuroinflammation. 2019 Apr 13;16:86. doi: 10.1186/s12974-019-1463-y (PMC6461821; doi:10.1186/s12974-019-1463-y)
Supplement: Supplementary file 1 — Table S1. Animals recruited in this study. Table S2. Primary antibodies used in study. Fig. S1. Astrocytosis and evidence of BBB breakdown observed with SIV infection Double IF staining for GFAP (red) and DAPI (blue) shows a significant increase in astrocyte coverage with SIV infection (a). Double IF staining for ZO1 (green) and DAPI (blue) shows a trending decrease in ZO1 expression with infection, but no significant difference (b). Triple-label IF of fibrinogen (red), Glut-1 (green), and DAPI (blue), shows a significant increase in the percent of vessels showing fibrinogen extravasation in SIVE animals when compared to uninfected (c). Linear analysis of MFI for both GLUT1 (green) and fibrinogen (red) provides an overlaid histogram view of the vessels in Figure S1c (d). Fibrinogen (red) that occurs outside the x-axis bounds of the two main GLUT1 (green) peaks is considered to be extravasated fibrinogen (d). Error bars indicate SD. Triple IF staining for vascular GLUT1 (green), pericyte CD146 (red) and nuclear DAPI (blue) shows a non-lesion-associated vessel (left) with pericyte coverage and a lesion-associated vessel (right) without pericyte coverage (e). (Docx 294 kb) [file 12974_2019_1463_MOESM1_ESM.docx]

**SUPPLEMENTARY MATERIAL**

***Journal of Neuroinflammation***

**Dysregulation of Sonic Hedgehog Pathway and Pericytes in the Brain after Lentiviral Infection**

Diana G. Bohannon^1^, Allen Ko^1^, Adam R. Filipowicz^1^, Marcelo J. Kuroda^2^, Woong-Ki Kim^1,^*

*^1^Department of Microbiology and Molecular Cell Biology, Eastern Virginia Medical School, Norfolk, Virginia, United States; ^2^Division of Immunology, Tulane National Primate Research Center, Covington, Louisiana, United States*

Correspondence: Woong-Ki Kim, PhD, Department of Microbiology and Molecular Cell Biology, Eastern Virginia Medical School, 700 W. Olney Road, Lewis Hall 3174, Norfolk, VA 23501, USA. Tel.: 757-446-5639. *E-mail:* [kimw@evms.edu](mailto:kimw@evms.edu)

**Table S1. Animals recruited in this study**

| Necropsy # | Age at Necropsy (y) | Infection Status | Degree of SIV Encephalitis |
| --- | --- | --- | --- |
| 11A554 | 10.28 | SIVmac251 | Mild SIVE |
| 11A562 | 5.15 | SIVmac251 | Mild SIVE |
| 13A400 | 5.96 | SIV0302-2 | Mild SIVE |
| 12A572 | 5.4 | SIV0302-2 | Mild SIVE |
| 11A201 | 10.68 | SIVmac251 | Severe SIVE |
| 10A067 | 5.7 | SIVmac251 | Severe SIVE |
| 11A837 | 8.28 | SIVmac251 | SIVnoE |
| 12A305 | 7.95 | SIVmac251 | SIVnoE |
| 12A602 | 6.31 | SIVmac251 | SIVnoE |
| 12A500 | 5.42 | SIVmac251 | SIVnoE |
| 11A014 | 4.77 | Uninfected | n/a |
| 11A313 | 5.02 | Uninfected | n/a |
| 11A635 | 5.41 | Uninfected | n/a |
| 11A023 | 4.69 | Uninfected | n/a |

n/a, not applicable

**Table S2. Primary antibodies used in study**

| Antibody | Clone | Ig Type | Manufacturer | Catalog | Use | Dilution |
| --- | --- | --- | --- | --- | --- | --- |
| Fibrinogen | Polyclonal | Rabbit IgG | Dako | A0080 | IHC/IF | 1:1000/1:100 |
| GFAP | MCA-5C10 | Mouse IgG1 | BioLegend | 801101 | IHC/IF | 1:1000/1:100 |
| GFAP | Polyclonal | Rabbit IgG | Abcam | ab7260 | IHC/IF | 1:2500/1:500 |
| GLUT1 | SPM498 | Mouse IgG2a | Thermo | MA1-37783 | IHC/IF | 1:2000/1:200 |
| GLUT1 | Polyclonal | Rabbit IgG | Thermo | RB-9052-P | IHC/IF | 1:1000/1:100 |
| PDGFrB | Y92 | Rabbit IgG | Abcam | ab32570 | IHC/IF | 1:400/1:20 |
| SHH | Polyclonal | Rabbit IgG | Boster | PA1072-1 | IHC/IF | 1:500/1:50 |
| SIVp28 | 3F7 | Mouse IgG1 | Fitzgerald | 10-002201 | IHC/IF | 1:1000/1:200 |
| ZO1 | Polyclonal | Rabbit IgG | Invitrogen | 40-2300 | IHC/IF | 1:400/1:80 |
| Netrin-1 | Polyclonal | Gt IgG | R&D Systems | AF1109 | IHC/IF | 1:400/1:40 |
| Fibrinogen | N/A | Ms IgG1 | Abcam | Ab58207 | IHC/IF | 1:1000/1:100 |

**Supplementary FIGURE 1**


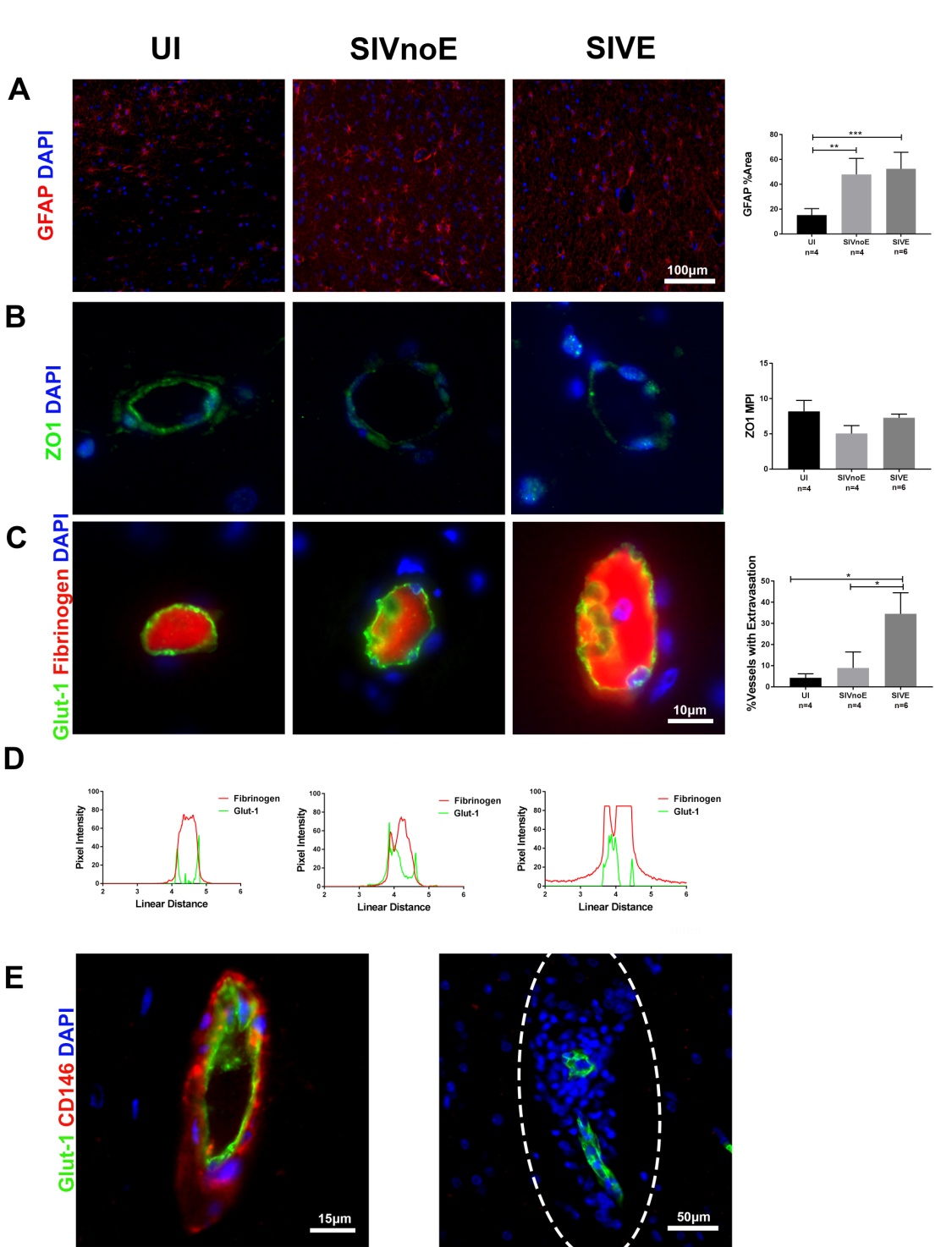


**Fig. S1 Astrocytosis and evidence of BBB breakdown observed with SIV infection** Double IF staining for GFAP (red) and DAPI (blue) shows a significant increase in astrocyte coverage with SIV infection (a). Double IF staining for ZO1 (green) and DAPI (blue) shows a trending decrease in ZO1 expression with infection, but no significant difference (b). Triple-label IF of fibrinogen (red), Glut-1 (green), and DAPI (blue), shows a significant increase in the percent of vessels showing fibrinogen extravasation in SIVE animals when compared to uninfected (c). Linear analysis of MFI for both GLUT1 (green) and fibrinogen (red) provides an overlaid histogram view of the vessels in Figure S1c (d). Fibrinogen (red) that occurs outside the x-axis bounds of the two main GLUT1 (green) peaks is considered to be extravasated fibrinogen (d). Error bars indicate SD. Triple IF staining for vascular GLUT1 (green), pericyte CD146 (red) and nuclear DAPI (blue) shows a non-lesion-associated vessel (left) with pericyte coverage and a lesion-associated vessel (right) without pericyte coverage (e).
